# Neurogenic Dysphagia and Nutrition in Disorder of Consciousness: An Overview with Practical Advices on an “Old” but Still Actual Clinical Problem

**DOI:** 10.3390/medicines9020016

**Published:** 2022-02-21

**Authors:** Loredana Raciti, Gianfranco Raciti, Grazia Pulejo, Valeria Conti-Nibali, Rocco Salvatore Calabrò

**Affiliations:** 1 GCA-Centro Spoke AO Cannizzaro, Catania, IRCCS Centro Neurolesi Bonino-Pulejo, 95122 Messina, Italy; loredana.raciti79@gmail.com (L.R.); gianfranco.raciti@gmail.com (G.R.); 2 Neurorehabilitation Unit, IRCCS Centro Neurolesi “Bonino Pulejo”, 98123 Messina, Italy; grazia.pulejo@irccsme.it (G.P.); valeria.continibali@irccsme.it (V.C.-N.)

**Keywords:** dysphagia, silent aspiration, nutrition, DOC, neurorehabilitation, FEES

## Abstract

Neurogenic dysphagia is a difficulty in swallowing food caused by disease or impairment of the nervous system, including stroke and traumatic brain injury. The most clinically apparent complication of neurogenic dysphagia is pulmonary aspiration, which may manifest itself acutely as choking or coughing, respiratory distress, wheezing, gasping or gurgling, and tachycardia. However, chronic symptoms, including weight loss, production of excessive oral secretions and aspiration pneumonia, may be also present, especially in patients with a disorder of consciousness (DOC). Usually, patients with dysphagia after the acute phase need to be treated with enteral nutrition using a feeding tube. This avoids patient malnutrition and supports the rehabilitation program. This narrative review aims to investigate dysphagia and its complications and management in patients with DOC. Clinical indications and practical advice on how to assess and treat this complex problem are also provided.

## 1. Introduction

Consciousness is considered an ambiguous term, encompassing both wakefulness (the state of being wakeful) and awareness (the state or level of consciousness where sense data can be confirmed by an observer) [1].

Chronic unconsciousness (DOC) is a tragic and ironic failure of high-technology treatment to preserve or restore brain function, which is the primary aim of therapeutics following brain injury. Usually, patients with DOC are affected by severe acquired brain injury (with a loss of consciousness lasting at least 24 h), including traumatic brain injury (TBI), devastating intracerebral haemorrhage and ischaemic stroke [2]. Based on the awareness, DOC can be classified as: (i) coma, i.e., the complete absence of arousal and awareness [3]; (ii) vegetative state (VS)/unresponsive wakefulness syndrome (UWS), which is defined as arousal without awareness [4]; and (iii) minimally conscious state (MCS), that is defined as slight varying awareness [5].

A patient in a VS appears at times to be wakeful, with cycles of eye closure and opening resembling those of sleep and waking. However, close observation reveals no sign of awareness or of a ‘functioning mind’. In detail, there is no evidence that the patient can perceive the environment or his/her own body or communicate with others, but the patient can breathe spontaneously and has a stable circulation.

The MCS is a condition of severely altered consciousness in which minimal but definite behavioral evidence of self or environmental awareness is demonstrated. MCS is characterized by behaviors associated with conscious awareness that occur inconsistently but are reproducible or sustained long enough to discern their nature [6,7].

Management of a patient with DOC requires carefully reaching the correct diagnosis, pronouncing an evidence-based prognosis, and thoughtfully considering the medical, ethical and legal elements of optimum treatment [8].

The main objective of this present review was to investigate swallowing disorders and oropharyngeal dysphagia in severe acquired brain injuries and to give some practical advice for the complex management of this devastating problem. 

Previous reviews have investigated the management of dysphagia in neurological disorders, mainly focusing on stroke [9] and paediatric populations [10]. To the best of our knowledge, only a previous work has specifically dealt with severe TBI [11]. However, our updated review focused on DOC, independently of the aetiology, and it aims to guide rehabilitation professionals in better managing dysphagia in a real clinical setting.

## 2. General Characteristics of Swallowing

Deglutition is the act of swallowing, through which a food or liquid is transformed into a bolus and brought into the stomach by voluntary and involuntary neuromuscular contractions. Normally, deglutition is performed in four phases: the first two (i.e., oral preparatory and buccal phases) are voluntary whereas the others (pharyngeal and oesophageal phases) are involuntary and based on peristaltic movements [12]. Then, we can classify swallowing as voluntary, spontaneous or reflexive, and emotional, in which swallowing movements occur during stressful conditions [13]. The neural control of swallowing involves many areas: from the motor nuclei in the brainstem to the cerebral cortices with complex sensory feedback. The network located in the brainstem is defined as the swallowing central pattern generator [13]. However, because of the various crossing controls of swallowing, ventilation and mastication functions in the brainstem areas, the control of these tasks is imbricated. The frontal, pre-frontal and parietal cortex are involved in the voluntary initiation of swallowing. Sensory stimuli coming from the mouth, pharynx and oesophagus, with the related ascending pathways, are fundamental to both voluntary and involuntary control. The central regulations define the elaboration and project of swallowing as well as the emotional stimuli of food intake.

## 3. Disorders of Swallowing

Swallowing disorders are frequent in DOC, with an incidence ranging from 25% to 61% [14]. Because dysphagia is often severe in this patient population, DOC usually needs artificial nutrition also in the acute phase, when nutrition and hydration are provided by the enteral-feeding tube [15,16]. Disorders of swallowing are under-diagnosed and often overlooked, although they are associated with severe complications, such as aspiration pneumonia or malnutrition. Then, dysphagia and the high rates of medical complications, which are present in two-thirds of DOC patients in both intensive care units and in rehabilitation wards [17,18,19], have an important impact on the prognosis and functional recovery. Indeed, this latter not only depend on the entity of the cerebral injury but also on the comorbidities and/or associated clinical conditions [20].

### 3.1. Dysphagia

Dysphagia is an anomalous swallowing due to an impaired coordination or impairment of swallowing biomechanics. This alteration may result in several complications, including dehydration, malnutrition, bronchospasm, and airway obstruction, as well as aspiration pneumonia and chronic chest infection. There are two events that may occur because of dysphagia: penetration and aspiration. Penetration occurs when liquid or bolus move into the laryngeal area to the level of the true vocal folds; aspiration occurs when the bolus or liquid penetrate below the trachea. Aspiration pneumonia represents the most common form of hospital-acquired pneumonia [19], and may be expressed by coughing. However, aspiration may occur without coughing, namely silent aspiration. Patients with previous documented aspirations had a total 10-fold increased risk of aspiration, and those with silent tracheobronchial aspirations had a 13-fold increased risk for developing pneumonia [20,21,22,23,24]. Patients with penetration can have a four-fold increased risk of pneumonia, as compared to healthy control subjects [22]. Since the mortality rates range from 20% to 65%, [24], swallowing disorders should be particularly addressed in patients with DOC receiving intensive rehabilitation. Oropharyngeal dysphagia affects between 50% and 78% of patients with stroke within the first week of admission, and this confers a three-fold increased risk of pneumonia [25]. Thus, when dysphagia persists, a gastrostomy tube for feeding is mandatory and its management requires behavioral adaptations (e.g., changing food consistencies, compensatory maneuvers, and biofeedback), neuromuscular-stimulation strategies and enteral feeding. Indeed, the evaluation of dysphagia required either an objective assessment of motor and sensitive functions of oro-buccal functions and cognitive and behavioural evaluation, using, e.g., the Levels of Cognitive Functioning scale (LCF). The LCF provides a simple way of systematically describing and categorizing a patient’s present level of consciousness and cognitive and behavioural functioning into one of eight levels through which DOC individuals typically progress during their stay in intensive rehabilitative care [26]. Then, an instrumental assessment, including a fiberoptic endoscopic evaluation of swallowing (FEES) or a videofluoroscopy (VFSS), is needed.

### 3.2. Characteristics of Dysphagia in Unresponsive Wakefulness Syndrome (UWS) or Minimally Conscious State (MCS)

The swallowing phases that are generally more damaged are the preparatory oral and pharyngeal phase, while the oesophageal phase is greatly spared. In a recent study, patients with DOC presented at least one kind of swallowing dysfunction. The authors showed that none of the VS patients was orally fed with solid or liquid food contemporarily, because of the presence of tracheostomy and/or the characteristic of dysphagia (absence of an effective swallowing oral phase and less effective pharyngeal phase), whereas the MCS group was not able to obtain ordinary food orally, probably due to the inability to reach a complete level of consciousness [27]. The presence of consciousness is the prerequisite for the effectiveness of the oral phase [28]. In fact, this phase is considered as the main voluntary (conscious) part of the swallowing process, controlled by several cortical regions that interact with the brainstem [29,30] and can produce masticatory-like movements and rhythmic tongue activity [31]. The cranial motoneuron groups of the hypoglossal, trigeminal, facial and vagal nerve are also involved [32].

This could explain why MCS present a partially functioning oral phase of swallowing, including lip prehension, lingual propulsion and no post-swallowing oral stasis, differently from UWS.

Usually dysphagia presents with incoordination, slow and imprecise swallowing acts, prolonged oral transit, depressed swallowing reflexes and reduced pharyngeal peristalsis. Between UWS and MCS there is a different spontaneous saliva management due to impairment of swallowing reflexes and ineffective pharyngeal phase of swallowing, as well as an impairment of cough reflex. This causes an increase in pharyngo-laryngeal secretions and saliva aspiration in UWS [27,33].

The typical condition of these patients is, therefore, the presence of different causes for dysphagia related to functional problems, organic central and peripheral neurological problems, as well as cognitive-behavioural alterations.

To summarize, dysphagia in these patients usually present and/or is related to the following different features:

- A functional disorganization of the motor patterns involved in swallowing, within a broader framework of sensory, motor and cognitive pathways.
- Central neurological brain stem lesions: responsible for reduced changes in motility, tone and sensitivity of oral and pharyngeal-laryngeal and absent/reduced swallowing reflex and cough.
- Partially reversible alterations, linked to the presence of the tracheostomy tube and the duration of assisted ventilation.
- Very low or fluctuating level of consciousness.
- Alterations and deficit of head and trunk control.
- Presence of archaic reflexes.
- Poor oral hygiene, with the appearance of mycosis and inflammatory conditions that may interfere with the swallowing mechanism.
- Facial or jaw fractures, tooth extraction, changes in the mandibular articulation as possible consequences of either the trauma or pro-tracheal intubation and prolonged maintenance of supine posture and half-open mouth during the acute phase.
- Peripheral neurological injury of the cranial nerves.

Furthermore, in patients with DOC many of the protective conditions for safe swallowing, including voluntary conscious activities such as raclage, voluntary coughing, swallowing on demand, maintaining a safe posture and the pneumo-phonic coordination, are not present. Nonetheless, in some patients it is possible to potentiate a pure swallowing reflex, in which the food in the mouth activates the process, in the absence of awareness or attention [27,34]. 

#### Dysphagia: Focus on in Severe Traumatic Brain Injury (TBI) 

The incidence of swallowing impairment in TBI ranges between 25% to 93% [35]: 51% of patients admitted with severe head injury showed pharyngeal problems affecting swallowing, and 31% showed behavioral problems affecting eating [36]. Management and treatment of these problems is important to avoid malnutrition, dehydration and aspiration pneumonia. 

The pathophysiological mechanisms subtending swallowing disorders in TBI are related to alterations of the oropharyngeal function, cognitive deficits and behavioral problems [36,37,38,39]. In particular, the main characteristics of dysphagia in TBI patients are: prolonged oral transit, delayed swallowing reflex and altered lingual control [40], as well as pharyngeal dysfunctions, including aspiration [39]. Independent predictors of impaired oral intake have been described in the literature, and are represented by the Rancho Los Amigo Scale, computerized tomography (CT) scans, ventilation time and aspiration [41].

About treatment measures of dysphagia in TBI patients, conventional treatments have been recommended. [42]. However, a specific effectiveness has not been shown. 

In a recent study, swallowing characteristics and the severity of dysphagia were studied and compared in TBI and stroke patients [43]. Radiologically, the lesion location was supratentorial in both groups (TBI, 92.7%; Stroke, 73.1%). TBI dysphagia was characterized by comprised aspiration or penetration, decreased laryngeal elevation and reduced epiglottis inversion. Nevertheless, no significant differences were found between the two patient groups.

Then, the conventional treatment for stroke patients could be applied also to TBI patients to improve pharyngeal transit time: change in posture and position for swallowing, learning new swallowing maneuvers, neuromuscular pharyngeal electrical stimulation, alteration in food amounts and texture and acupuncture [42,44]. 

### 3.3. Assessment of Dysphagia

The evaluation of swallowing disorders consists of a general and clinical swallowing (“bedside”) examination and the use of dynamic imaging techniques [45]. The procedures are interrupted if individuals aspirate more than 50% of the bolus on three consecutive swallows. Contemporarily, penetration-aspiration scores on VFS can be scored offline by two speech and language therapists [46]. The evaluation protocol of dysphagia is applied as soon the patient is hospitalized in the intensive rehabilitation unit, and requires the use of the methods chosen according to the degree of consciousness of the patient [47]. In fact, given the low/no cooperation of the patient, the clinical assessment tools are initially observation and stimulation. The clinical evaluation of dysphagia is usually performed by a speech pathologist trained in the management of dysphagia, and a neurologist. This usually includes a bedside evaluation with the assessment of motor function and signs and symptoms of dysphagia, including the presence of pneumonia, unjustified weight loss, pulmonary status, current method of nutrition and nutritional status. The patient’s behavioral characteristics, with particular attention to the level of alertness, are always taken into consideration. Clinical examination should address the labial competence, tongue motility and soft palate, the extent of drooling and areal space, cough, and gag reflex to breathing pattern [48]. 

In a second moment, there is the administration of small amounts of still water and gelled water, which is indicated for the low risk of inhalation and subsequent complications. The addition of methylene blue is sometimes required. The evaluation of symptoms such as cough and voice quality (if emitted) and the possible detection of changes in oxygen saturation in the blood using transcutaneous oximetry are signs of dysphagia [49]. During the evaluation, it is possible to observe the appearance of automatisms, such as mouth-opening, closure of the lips around a teaspoon, triggering of deglutition, the appearance of other deglutition reflexes and lingual movements. This assessment cannot exclude the presence of silent inhalation. The test with methylene blue (blue dye test) has a relatively high proportion of false negatives and is not able to exclude micro inhalation. Therefore, to improve the reliability of bedside evaluation (BSE), some authors have tried to correlate oxygen saturation with the water swallowing test. A decrease of more than 2% in saturation is still considered sufficient to suggest a swallowing problem, so that the subject must be subjected to more accurate diagnostic evaluations, while a decrease of more than 5% must immediately lead to the interruption of the test because inhalation has occurred [49]. The tests of water swallowing, and desaturation performed in combination, provide a better measurement of both sensitivity/specificity, and positive and negative predictive value in detecting inhalation. The positivity of these tests necessitates a detailed investigation using imaging techniques, such as VFS and/or FEES that provide direct visualization of the anatomy and physiology of swallowing during deglutition. 

**Figure 1:**
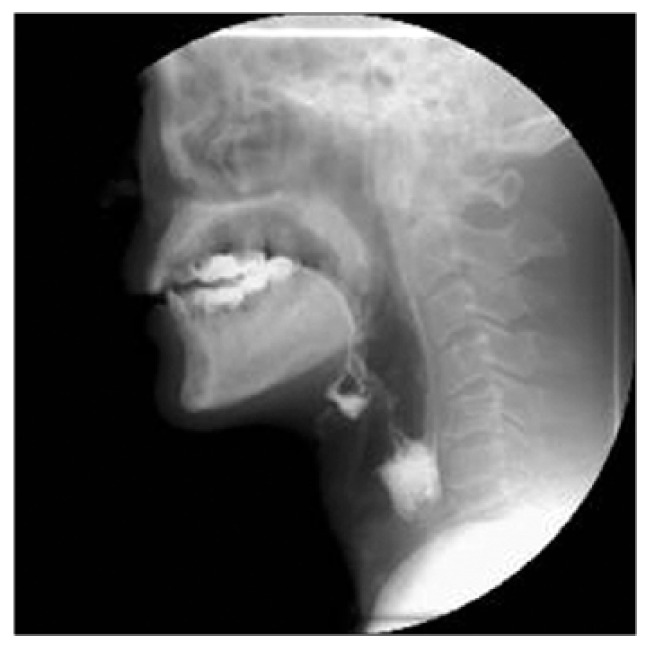
A typical radiogram of the videofluoroscopy (VFS).

The VFSS (Figure 1) is a radiograph of the bone, cartilage, and soft tissue swallowing structures visualized while the food and liquid mixed with barium passes through all stages of the swallow [50]. Videoendoscopic investigation allows a static and dynamic assessment of the structures of the upper respiratory and digestive tract. The static evaluation, aside from giving information of the anatomical structures involved in swallowing, allows the function of the laryngeal sphincter to be studied and the possible detection of stagnation; dynamic evaluation by the administration of a bolus, allows proper swallowing function to be evaluated. 

**Figure 2:**
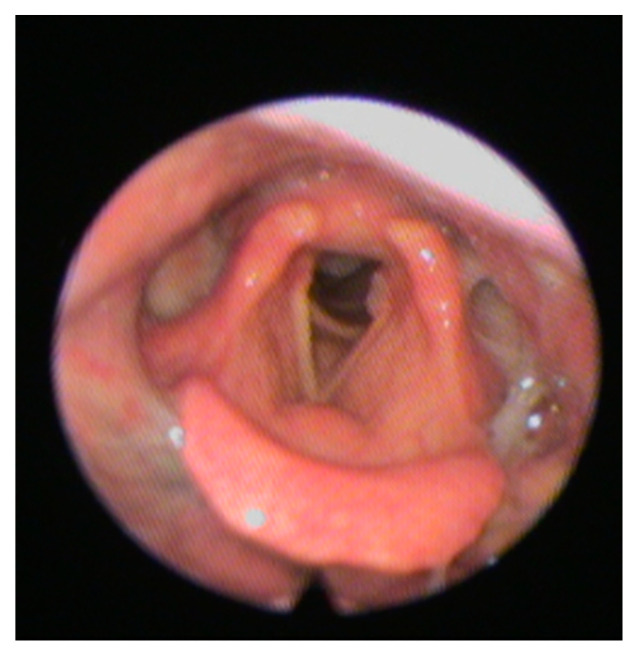
Shows a typical fiberoptic endoscopic evaluation of swallowing (FEES) image.

The FEES is a transnasal passage of a flexible nasopharyngoscope to provide direct observation of the pharynx and larynx before and after the swallow [51]. This latter is preferable in cooperative patients with suspect silent aspiration and is practical especially in cases where there is uncertain inhalation or contributing factors, such as a risk of cough ineffectiveness. FEES allows a good view of stagnation in the valleculae, in the pyriform sinuses, in the laryngeal vestibule (Figure 2).

Furthermore, stimulation with the tip of the endoscope permits to trigger the pharyngeal and laryngeal zones. 

Nonetheless, both methods give reliable results only in patients with LCF ≥ 4. Based on a recent consensus of speech pathologists, an instrumental assessment of swallowing should be performed in patients with DOC, and FEES is usually better tolerated than VFSS because of the possibility to perform FEES at the bedside using food, detecting aspiration, and avoiding radiation [34]. A recent review underlines the importance of using FEES [52,53] or even a VFSS [46], since patients diagnosed by means of these two instruments have better oral intake outcomes than those who do not. Then, a systematic evaluation of swallowing should be performed to guide clinical decisions about oral intake and not rely on level of consciousness [28,54]. Although FEES has been suggested as the gold standard in dysphagia assessment and management [55], further studies are needed to better understand its use as well as the pathomechanisms of swallowing in patients with DOC. Moreover, the use of appropriate scales, i.e., the Swallowing Rating Scale and the DOSS-dysphagia Outcome and Severity Scale of O’Neil (see Appendix A and Appendix B), are necessary to improve the functional prognosis of the patients as well as ensure the appropriate treatment and planning of a therapeutic path to follow (weaning from aids such as PEG, the SNG and the tracheostomy tube), especially for patients with nasogastric tube. 

Mélotte et al. published a validation protocol of a swallowing assessment tool for patients with DOC, called SWADOC. This tool allows us to objectively evaluate the efficacy of therapy, and to provide a clear and accurate summary of the patient’s limits favoring dysphagia-oriented therapy [55,56,57]. Finally, the Functional Oral Intake Scale (FOIS), a reliable tool to assess functional oral intake of food and liquids [57], could support the diagnosis.

### 3.4. Rehabilitation of Dysphagia

The goal of treatment of patients with cough due to oral-pharyngeal dysphagia is to avoid aspiration pneumonia. The guideline of the American Gastroenterological Association on oral pharyngeal dysphagia management, due to its complexity, recommends a multidisciplinary approach, consisting of a neurologist, a nurse, occupational, physical, and speech therapists, a pharmacist, and a dietitian. One of the widely used approaches in neurorehabilitation is the Facial Oral Tract Therapy (F.O.T.T.^R^), a structured issue to evaluate and treat patients with disturbances in swallowing and eating, oral hygiene, non-verbal communication, and speech articulation caused by neurological diseases [58,59,60]. The tool includes the use of compensatory strategies, such as postural adaptations, sensory stimulation, manipulations of volume, consistence and viscosity of food.

The interventions can be categorised into the two main treatment modalities, that is cortical or non-cortical stimulation of the swallowing network [61].

#### 3.4.1. Non-Cortical Stimulation

Non-cortical interventions are usually conventional treatments increasing sensory input to the swallowing network in the brain, and potentiating the activity of the motor swallowing areas in the cortex, brain stem, and related neural networks [61]. Six categories were defined: (i) complex swallowing interventions, (ii) neuromuscular electrical stimulation (NMES), (iii) pharyngeal electrical stimulation (PES), (iv) sensory stimulation (including sensory electrical stimulation (SES), (v) thermostimulation and thermal/tactile stimulation, and (vi) strengthening exercises and respiratory muscle training.

The conventional treatment of dysphagia consists of exercises to increase oropharyngeal muscles strength as well as compensation strategies (including positioning, posture change and dietary modification to promote swallowing physiology and increase sensory input through thermal–tactile stimulation) [62]. However, as the aforementioned outcomes could be reached at different times by the different patients, personalized treatment has to be considered [58,59,60]. UWS patients with complete dysphagia and enteral feeding, as well as MCS patients with severe dysphagia or mixed feeding (enteral and oral), should be treated with peri-buccal and oral sensitivity stimulation by thermal-tactile-vibratory, passive neuromotor treatment and/or active orofacial structures (massage, praxis), stimulation of the swallowing reflex, exercises to restore the costo-diaphragmatic breathing with particular attention to the stimulation of cough and apnea mechanism, and management of oral secretions to facilitate the recovery of swallowing function (remedy methods) [61]. Moreover, in MCS patients attempts toward swallowing with modified food in small quantities (ice cream, water gel) as well as other food consistencies (mousse, creams) should be made. After the different treatments, most patients with DOC reaches the possibility of partial oral nutrition by recovering oral automatisms that allow effective swallowing and, therefore, the possibility to introduce sufficient daily nutritional supplements [27]. This is generally accomplished using only creamy and homogeneous consistencies combined with dietary management, postural compensation, swallowing manoeuvres, change in the food consistency, choice of the most suitable bolus volume, attention to temperature and taste. The rest of the diet is completed by the contribution provided by the PEG [63,64].

Some studies evaluated the acupuncture as an add-on to a conventional therapy with significant results at 4 weeks follow-up [65]. Few descriptive case studies observed the results of F.O.T.T., using principles for motor learning, increased oral intake and improved safety of swallowing [66,67]. Neurophysiological stimulation has been recently applied to improve conventional treatment results [68]. A new treatment option using neuromuscular electrical stimulation (NEMS) has recently been introduced. This, combined with the traditional speech therapy treatments for swallowing, showed good potential for significant and lasting improvement of dysphagia [69,70]. The targeted treatment for dysphagia through the use of NMES involves the application of electrodes to the skin of the head and neck in order to obtain, thanks to the pulses of electrical energy, a stimulation of the muscles that are weakened or paretic. Then, the strengthening of the oropharyngeal musculature improves swallowing physiology [68,69,70,71]. The use of NMES in the treatment of dysphagia has revolutionized the way of thinking in the field of deglutition rehabilitation. Since the beginning of the method many controversies were born as regards the real clinical application of the NMES protocol for swallowing. Moreover, even the fact that the same treatment was proposed for patients suffering from a wide range of disorders and the need to train health care professionals to its use have stirred much debate within the scientific community. Although this technique may contain rehabilitative potential for certain groups of patients, especially for those with stroke [72], the widespread use of this technique does not mean that it is synonymous with a universal rehabilitative and equally effective approach for dysphagia. Indeed, the results about NMES are inconsistent and still controversial. 

As with the NMES, the PES aims to strengthen the impaired oropharyngeal musculature targeting the peripheral neuromuscular system. Hamada et al. studied surface PES in combination with general dysphagia therapy, stimulating the mylohyoid muscle with an amplitude of the electrical current set to the sensory threshold level, and inducing neuroplastic changes in the sensory cortex. Results showed a positive effect on procedures of decannulation [73]. However, Bath et al. found significant improvement from baseline to three-month post-PES treatment on the DOSS for 20 patients in patients with TBI [74]. Prosiegel et al. sought to trigger the swallowing reflex through thermo-stimulation obtaining positive changes in oral intake and decannulation [75]. One study tested an intervention of cervical strengthening exercises against resistance in four directions with improvement in oral intake at the end of treatment (12 weeks) [76]. Another study investigated an oral device (Muppy) for oral neuromuscular training aimed at stimulating sensory input and strengthening the facial, oral, and pharyngeal muscles resulting in improvement of swallowing at 1 year [77]. On the other hand, respiratory muscle training did not show differences with a control group [78]. The difficulties that prevent a complete oral intake may be due to a compromised oral motor deficit and/or from some persistent pathological reflexes together with serious disturbances of consciousness that slow the timing and the amount of intake [13]. Moreover, oral feeding is encouraged both for its hedonistic value and because meals are an important opportunity for relationship and communication between patients and their families, and for the prevention of secondary damage to oral mucosa, trophic muscle, teeth and temporo-mandibular joint.

#### 3.4.2. Cortical Stimulation

Non-invasive brain stimulation (or cortical stimulation) consists of repetitive transcranial magnetic stimulation (rTMS), that modulates cortical excitability by focally stimulating the cortical motor areas associated with swallowing, and transcranial direct current stimulation (tDCS). All studies about rTMS varied in modes and Hz of stimulation. The studies showed a better significant improvement of the outcomes when rTMS was combined with conventional therapy [61]. The non-invasive tDCS is a cortical stimulation technique aimed to project the pharyngeal representation to the unaffected hemisphere, hypothetically ensuring increased input to the brainstem swallowing centres. The integrity of brainstem is fundamental [79,80]. However, only one study showed an improvement in DOSS in the tDCS group compared with sham and a recent review underlined the low-quality evidence of the studies that could show the effectiveness of non-invasive brain stimulation in improving dysphagia after acquired brain injury [81].

## 4. Artificial Nutrition: Clinical Indications

People affected by DOC are usually treated with artificial nutrition even in the acute phase of the disease. Indeed, when these patients attend the Neurorehabilitation Unit, the use enteral nutrition (EN) may persist and/or is indicated, because of the presence of severe dysphagia, which causes malnutrition and aspiration pneumonia with morbidity and mortality, especially in patients with tracheostomy [82,83].

Then, dysphagia and high rate of medical complications during treatment of DOC patients in both the intensive care unit and in rehabilitation ward have an important impact on the prognosis and functional recovery that not only depends on the entity of the cerebral injury but also on the comorbidities and/or associated clinical conditions [17,18,19,20]. These patients are at high risk for developing diseases, such as pulmonary and urogenital infections, due to bed riddance that can, therefore, undermine the rehabilitative potential and increase overall hospitalization. They often suffer of metabolic imbalance, but artificially supplied nutrients can reverse catabolism, preserve and promote the integrity of the immune status thus reinforcing organism resistance. Adequate nutrition is therefore important in order to avoid complications, reduce hospitalization time, improve quality of life, and make the therapeutic path simpler and more effective [84,85,86].

This is why EN is an integral part of the therapeutic approach and plays an important role in the outcome of the patient. EN is the choice treatment as it is provided through the physiological gastrointestinal system, thus favoring the trophism and microbial diversity of the intestinal mucosa. Moreover, from a metabolic point of view, it is better tolerated and has a lower cost than total parenteral nutrition [87,88]. 

Parental nutrition (PN) is the administration of energy substrates through a central venous catheter. This is indicated when it is not possible to maintain adequate nutrition, fluids, and electrolytic balance through oral or enteral nutrition for a period exceeding 14 days or in case of non-availability of a peripheral venous access. However, the PN is usually associated with more infectious complications, related to increased alimentation and hyperglycemia [20]. 

Elke et al. in a recent review and meta-analyses of 18 randomized controlled trials evaluate the effect of EN versus PN on clinical outcomes and showed that there was no difference in mortality and mechanical ventilation between the two modalities. Nonetheless, a reduction on the hospital length of stay and on the incidence of infectious were reported in the EN group, likely due to the preservation function of gut and intestinal microbial diversity as well as gut-mediated immunity [87]. 

This could be explained by the different amount of caloric intake. The higher risk of infectious of PN nutrition has been related to the excessive macronutrients in an impaired metabolic control of an early phase of the illness [88]. Indeed, the hyper caloric intake is considered a negative outcome itself because it leads to an increased risk of infectious complications [88,89].

Then, according to the recent guideline recommendations, the EN should be considered the first-line treatment in DOC patients [90]. Concerning the correct caloric intake, based on the above consensus, the energy intake should be evaluated based on the determination of the indirect calorimetry (IC) or a weight-based equation (25–30 kcal/kg/d). In particular, proteins should be evaluated as the most important macronutrient because of their function in healing wounds, supporting immune function, and maintaining lean body mass. Then, the amount of proteins has to be calculated by the weight-based equations and has to be in the range of 1.2–2.0 g/kg/d [90].

Artificial nutrition is of primary importance in ensuring adequate nutrition for the prevention of protein and caloric deficit and to prevent metabolic deterioration as well as loss of body mass responsible for the highest increase in therapeutic failure, complications and mortality. The first approach of the nutritionist, within 24–48 h from admission to the Intensive NeuroRehabilitation ward is the assessment of the nutritional status and subsequent monitoring of the patient. The evaluation should begin with the nutritional anamnesis to highlight the presence of other diseases and/or treatments that may result in increased needs for energy and nutrients or request changes in the nutritional composition of the diet. The oral intake of foods cannot be assessed in such patients, and it is considered inappropriate and therefore risking nutrition where input is generally lower than 50% of the overall needs for a period greater than 7–10 days. The initial evaluation includes a careful examination and detection of anthropometric parameters. Among them, weight, height and BMI are important predictors of mortality in patients hospitalized for serious conditions, whereas visceral proteins do not constitute a specific indicator of nutrition since they can depend on several factors. The evaluation of the immunological status through total lymphocytic count could be also of help. Repeated body weight measurement should be carried out at least weekly for the entire duration of hospitalization. Unintentional weight loss (clinical sign of negative caloric protein balance) of 5% in 3 months or 10% compared to normal weight before illness in the last 6 months is considered a risk factor for malnutrition. The impedance with the new piezoelectric soft tissue analyzer (STA) is a non-invasive, rapid and objective analysis capable of directly identifying cell mass in Kg and percentage content of extracellular water. 

Adequate nutrient supply is indicated by an increase in weight, oedema disappearance, increased muscle strength and work capacity, improved appetite and a sense of well-being. Other biochemical assessments are the knowledge of vitamins and trace elements status, protein balance and protein metabolic rate, and the evaluation of delayed hypersensitivity reaction (skin test) [91,92].

## 5. Practical Advices on Dysphagia Management

**Figure 3:**
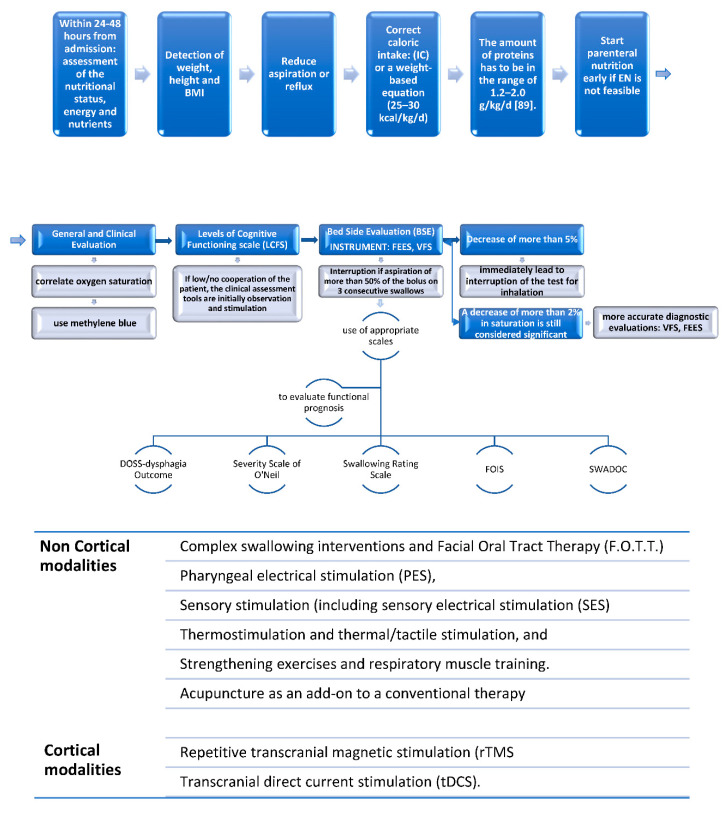
The three-step algorithm to manage DOC dysphagia; Legend: IC: indirect calorimetry; EN: enteral nutrition; FEES: fiberoptic endoscopic evaluation of swallowing; VFS: videofluoroscopy; SWADOC: swallowing assessment tool for patients with DOC; FOIS: Functional Oral Intake Scale.

Management of dysphagia is often a challenge when dealing with DOC in everyday clinical practise, and clear guidelines are useful to better face this important issue (Figure 3).

First, it is necessary to evaluate the general clinical conditions of the patient, assessing the nutritional status and the need for energy and nutrients. In fact, a careful examination and detection of anthropometric parameters (weight, height and BMI), that are predictors of mortality, are important. The first-line treatment in DOC patients is represented by enteral nutrition [90]. It is important to provide the patient with an optimal amount of proteins (range of 1.2–2.0 g/kg/d), because of their function on healing wounds, supporting immune function, and maintaining lean body mass. An adequate nutrient supply is indicated by an increase in weight, oedema disappearance, increased muscle strength and work capacity, improved appetite and a sense of well-being. 

In order to start deglutition training, the use of LCF is recommended, since, in our opinion, training should be tailored to the patient’s level of consciousness. UWS patients cannot be trained to actively potentiate their oral phase, and to search for effective swallowing reflexes is the main goal in this patient population. In MCS, the training should indeed be performed when cognitive abilities are more present. The evaluation of swallowing disorders should start with a detailed neurological and local clinical examination. This should address motor function and cranial nerves, including the labial competence, tongue motility and soft palate, the extent of drooling and areal space, cough, and gag reflex to breathing pattern.

The presence of moderate to severe dysphagia can be predicted in the presence of at least two out of the following six clinical signs: loss of voluntary cough and gag reflex, dysphonia, dysarthria, coughing, or changes in the voice quality after swallowing. 

The BSE examination could improve its sensitivity/specificity by using the oxygen saturation: a decrease of more than 2% in saturation is considered sufficient to submit the patient to more accurate diagnostic evaluations, while a decrease of more than 5% must immediately lead to interruption of the test for inhalation. Assessment should then be based on more specific clinical tools, such as the Swallowing Rating Scale and the DOSS, useful to both diagnosis and prognosis, and instrumental assessment, including FEES and/or VFSS. Nonetheless, in clinical practise VFSS may be rarely performed in these patients, and therefore FEES is to be considered the gold standard of dysphagia diagnosis.

Regarding the treatment, a multidisciplinary approach, including -but not limited to- a neurologist, a nurse, occupational, physical, and speech therapists, is mandatory. The two main treatment modalities are: cortical or non-cortical stimulation of the swallowing network (Figure 3). However, non-invasive brain stimulation is not widely available, and it is mainly used for research purposes. Thus, we recommend focusing on more easy approaches, as they are available in all of the clinical settings where skilled healthcare professionals are present. 

## 6. Conclusions

This narrative review provides simple and useful information on the characteristics and management of dysphagia in patients with DOC, highlighting the need for an accurate assessment to avoid malnutrition and other dysphagia-related medical complications. Instrumental investigation using VFSS and FEES is fundamental to objectively assess both structural abnormalities and motility disorders of the oropharynx. Then, an intensive and specific swallowing rehabilitation, as well as an adequate artificial nutrition, is necessary to improve the functional outcomes and quality of life of both patients with DOC and their caregivers.

## Appendix A. Swallowing Rating Scale

The logopaedic evaluation of the clinical and functional alteration of swallowing, with the application of bedside swallowing evaluation (BSE) and fiberoptic endoscopic evaluation of swallowing (FEES), identifies the degree of dysphagia according to the Swallowing Rating Scale (American Speech-Language-Hearing Association, ASHA, 2003), which provides a score between 1 and 7 (from 0 to 2 will usually refer to patients in a vegetative state (VS) or minimally conscious state (MCS): (ASHA). (2003).

**Table A1:** Shows the levels of swallowing rating scale.

Level 0	The patient cannot be evaluated.
Level 1	swallowing is not functional (SNG o PEG) *****.
Level 2	swallowing is inconsistent or delayed, prevents an adequate nutritional intake, although swallowing is sometimes possible. Modified consistency of food
Level 3	the alteration of swallowing partially prevents nutritional intake and requires close monitoring of the patient during feeding
Level 4	the alteration swallowing does not prevent nutritional intake, but supervision is required for the use of compensatory techniques.
Level 5	altered swallowing is sufficient for nutritional intake, but compensatory techniques are required and sometimes special nutrition techniques and dietary modifications.
Level 6	swallowing is functional for most of the feeding activity but sometimes there are difficulties. The meal can last longer
Level 7	Normal swallowing.

* SNG: Nasogastric Tube; PEG: percutaneous enterogastrc tube.

## Appendix B

The DOSS scale identifies the following levels of deglutition alteration:

Oral nutrition (PO): normal diet (level 6–7).

Oral nutrition: modified diet-level of independence substantially changed (level 5–3).

Oral nutrition is not necessary (level 1–2 are related to patients in vs. or MCS).

**Table A2:** Shows the levels of the DOSS Scale.

Full per os nutrition (P.O): Normal diet	Level 7 Normal	Normal diet No strategies or rehab needed
	Level 6 Minor swallowing alterations	Normal diet, functional swallow Patient may have slight oral or pharyngeal delay, or slight retention or trace epiglottal undercoating but spontaneously rewards May need extra time for meal No aspiration or penetration with any food consistency
Full P.O: Modified diet and/or independence	Level 5 Mild dysphagia: Remote supervision, may need one diet consistency restricted	Mild oral dysphagia with reduced mastication and/or oral retention that is cleared spontaneously: Aspiration of thin liquids only but compensate by valid reflexive cough Airway penetration midway to cords with one or more consistency or to cords with one consistency but clears spontaneously Retention in pharynx that is cleared spontaneously
	Level 4 Mild–moderate dysphagia: minimal Supervision, need command, one or two consistencies restricted	Retention in pharynx cleared with command Retention in the oral cavity that is cleared with cue Aspiration with one consistency, with weak or no reflexive cough Or airway penetration to the level of the vocal cords with cough with two consistencies Or airway penetration to the level of the vocal cords without cough with one consistency
	Level 3 Moderate dysphagia: Total assist and supervision, two or more diet consistencies restricted. Nonoral nutrition necessary	Moderate retention in pharynx, cleared with command Moderate retention in oral cavity, cleared with cue Airway penetration to the level of the vocal cords without cough with two or more consistencies Or aspiration with two consistencies, with weak or no reflexive cough Or aspiration with one consistency, no cough and airway penetration to cords with one, no cough
	Level 2 Moderately severe dysphagia: Maximum assistance or use of strategies with partial P.O. only	Severe incontinence or retention in pharynx, unable to clear or needs multiple cues Aspiration with two or more consistencies without reflexive cough, poor voluntary cough Or aspiration with one or more consistency, no cough and airway penetration to cords with one or more consistency, no cough Can tolerate only one consistency with the use of compensation postures or other swallowing techniques
	Level 1 Severe dysphagia: NPO: Unable to tolerate any P.O. safely	Severe retention in pharynx, unable to be eliminated Severe oral stagnation bolus or retention, unable to clear Silent aspiration with two or more consistencies, ineffective voluntary cough Or unable to swallow

* [93].
